# d-Serine’s Journey Between Stars and Synapses

**DOI:** 10.1007/s11064-025-04564-y

**Published:** 2025-10-14

**Authors:** Sarah Mountadem, Stéphane Henri Richard Oliet, Aude Panatier

**Affiliations:** Univ. Bordeaux, INSERM, Neurocentre Magendie, U1215, F-33000 Bordeaux, France

**Keywords:** Astrocyte, d-serine, Long-term synaptic plasticity, NMDA receptors, Tripartite synapse

## Abstract

Astrocytes play a pivotal role in regulating synaptic transmission, with d-serine emerging as a key gliotransmitter shaping NMDA receptor-dependent functions. This review is focusing on the multifaceted role of astrocytic d-serine from synaptic transmission to cognitive processes. While this review includes the work of other groups, it is mainly based on the findings obtained in our laboratory. Drawing from two decades of research spanning from the hypothalamus to the hippocampus, we here highlight how astrocyte-derived d-serine regulates NMDAR activity, long-term synaptic plasticity, and associated memory. Our findings have revealed the dynamic control exerted by astrocytic processes onto d-serine availability within the synaptic cleft, including the impact of the astrocytic morphological plasticity, the key role played by intracellular Ca^2+^ as well as the involvement of CB1 and EphB3 receptors. We also discuss how an impairment in astrocytic d-serine synthesis can affect the co-agonist availability and consequently impact cognitive functions in neurodegenerative disorders such as Alzheimer’s Disease. To conclude, this review highlights the role of astrocytic d-serine in astrocyte-neuron communication and higher-order brain functions.

## Introduction

For decades astrocytes were considered passive elements of the central nervous system, primarily providing structural and metabolic support to neurons. However, this traditional view has been significantly revised thanks to groundbreaking research revealing the active and dynamic roles played by astrocytes in neuronal communication. Among the pivotal figures in this transformation, Giorgio Carmignoto made enduring contributions by demonstrating that astrocytes detect, and in turn regulate, neuronal excitability and synaptic transmission through Ca^2+^ signaling and gliotransmission, respectively [1–4]. His pioneering work on astrocyte-neuron interactions laid the foundation of the tripartite synapse in which astrocytes play an active role in chemical transmission alongside pre- and postsynaptic neurons.

At the beginning of the twenty-first century, the work carried out in our laboratory on the hypothalamic supraoptic nucleus has shed light on the key role played by astrocytes in regulating synaptic transmission and plasticity. This brain area is known to undergo a striking and reversible anatomical remodeling under conditions where oxytocinergic magnocellular neurons are strongly activated, like during parturition and lactation. Such a remodeling is characterized by a significant reduction of the astrocytic coverage of oxytocin neurons and of the synapses impinging upon these neuroendocrine cells. We took advantage of this unique physiological model to study the impact of the astrocytic environment onto synaptic transmission and plasticity [5]. Among the different processes we investigated, we focused on d-serine, a co-agonist of glutamatergic N-methyl-d-aspartate receptors (NMDARs) [6], that is synthesized and released by astrocytes [7–10]. We investigated the consequence of this physiological glial anatomical remodeling occurring during parturition and lactation on NMDARs functions, including the most common forms of synaptic plasticity in the brain: NMDAR-dependent long-term potentiation (LTP) and long-term depression (LTD). We were able to show that the astrocytic environment of glutamatergic synapses is a key determinant for NMDAR activity through the release of d-serine [11]. This finding was proven to be true later on, at other central synapses, including at hippocampal CA3-CA1 synapses where NMDAR activity and synaptic plasticity have been extensively studied and documented [12, 13]. These insights not only underscore the central role of astrocytes in cognitive processes but also extend Carmignoto’s work by revealing how astrocytic signaling governs synaptic plasticity.

In this review, we are mainly summarizing key findings from our research over the past two decades, focusing on astrocytic d-serine and its role in brain functions, from synaptic transmission to cognition.

## Astrocytes Control the Activity of Synaptic NMDARs in the Hypothalamic Supraoptic Nucleus

Classical glutamatergic NMDARs are composed of two GluN1 and two GluN2 subunits and require the binding of two ligands to be activated: Glutamate that binds on the GluN2 subunit, and a co-agonist, that binds on the GluN1 subunit [6, 14]. For a very long time, glycine has been considered to be the only endogenous co-agonist of NMDARs. When glycine was first identified [14], other amino acids like d-serine were also found to bind the co-agonist site. However, the putative role of this d-amino-acid as an endogenous ligand was ruled out based on the assumption that it could not be synthesized by the mammal brain. This assumption was proven wrong several years later with the demonstration that d-serine not only can be synthesized in the brain but also released by astrocytes [8, 9]. Most importantly, endogenous d-serine was found to be important for NMDAR activity including LTP induction at hippocampal CA3-CA1 synapses [7, 10]. The exact role played by astrocytes in this regulation of NMDAR and synaptic plasticity was completely unaddressed at that time. To tackle this issue, we took advantage of the anatomical remodeling occurring in the supraoptic nucleus of lactating rats and investigated the consequences of the reduced astrocytic coverage of oxytocin neurons on NMDAR activity [11]. First, we performed HPLC, immunohistochemistry and confocal microscopy experiments to identify whether d-serine was synthesized in the supraoptic nucleus of the rat hypothalamus. While d-serine was found in the supraoptic nucleus of virgin animals, serine racemase, the enzyme converting l-serine into d-serine, was also expressed in astrocytes. Then, recordings of supraoptic neurons were performed in acute hypothalamic slices obtained from virgin and lactating rats to study synaptic NMDARs activity. We first assessed the nature of the endogenous co-agonist gating NMDARs in this region. To this end, we used d-amino acid oxidase (DAAO) or glycine oxidase (GO), two selective enzymes degrading respectively d-serine and glycine. While GO had no effect, the application of DAAO impaired significantly NMDARs activity, an effect that was rescued by the application of exogenous d-serine. These experiments demonstrated that d-serine, and not glycine, is the endogenous co-agonist of synaptic NMDARs in the rat supraoptic nucleus [11]. We next investigated the ability of glutamatergic synapses impinging upon supraoptic neurons to express NMDAR-dependent long-term plasticity. Strikingly, while both LTP and LTD could be readily induced in acute slices from the brain of virgin rats, the same was not true in slices from lactating rats. We found that it was more difficult to experimentally induce plastic changes at glutamatergic synapses under conditions where the glial coverage was reduced. Most interestingly, supplementing slices from lactating rats with exogenous d-serine completely restored the ability of glutamatergic synapses to express LTP and LTD. By releasing d-serine, astrocytes thus control the activity of postsynaptic glutamatergic NMDARs. The physiological anatomical remodeling that occurs during lactation modifies the co-agonist supply to these receptors, thereby impacting the direction and the magnitude of long-term synaptic plasticity [11]. These results demonstrated a critical role for astrocytes in the regulation of synaptic strength and introduced a new paradigm in which gliotransmission actively contributes to memory-related synaptic processes. The next step in investigating d-serine gliotransmission was to establish whether astrocytes are playing a similar a role in other brain regions and in particular in the hippocampus, a key brain structure in which NMDARs, and consequently NMDAR-dependent plasticity, are essential for learning and memory processes. At that time, it was known that the enzymatic degradation of d-serine in hippocampal acute brain slices, led to NMDARs activity and LTP impairments [7, 10]. Another group provided evidences that the occupancy of the NMDAR co-agonist binding site was not saturated under baseline conditions, suggesting that NMDARs are potential spatiotemporal detectors of afferent activity [15]. Therefore, as astrocytes also detect afferent activity and regulate synaptic transmission in a Ca^2+^-dependent manner, we then decided to investigate whether d-serine availability in the synaptic cleft was an astrocytic Ca^2+^-dependent process.

## d-Serine Release from Hippocampal Astrocytes is Ca^2+^-Dependent

In order to test whether d-serine release from astrocytes at CA3-CA1 synapses was Ca^2+^-dependent, individual astrocytes were loaded with a solution clamping intracellular free Ca^2+^ at a steady-state concentration of 50–80 nM [12]. Such Ca^2+^-clamping caused a significant decrease of NMDARs evoked responses at synapses located withing the territory of the dialyzed astrocyte. This impairment was rescued by providing d-serine to the tissue. It was also prevented when d-serine was added in the bathing solution from the beginning of the experiments. Not surprisingly, LTP was also impaired under conditions where astrocytes were dialyzed with the Ca^2+^-clamp solution, an effect that was also rescued by supplementing the tissue with exogenous d-serine. In this study, we went a step further by showing that LTP was impaired by dialyzing individual astrocytes with the light-chain of the tetanus toxin (LC-TT), suggesting that at least part of d-serine release is mediated via a vesicular pathway. As the LC-TT could not go through gap junctions, and therefore diffuse in the syncytium, we notice that LTP remained unaffected at synapses impinging upon neurons present in the anatomical territory of neighboring astrocytes that have not been dialyzed. This suggested that each astrocyte control NMDAR activity and coordinate plasticity within its own anatomical perimeter [12]. These findings not only confirmed the active role played by astrocytes in controlling NMDARs activity, and therefore the induction of synaptic plasticity, but also established astrocytes as spatially localized regulators of Hebbian plasticity. While our findings indicate that d-serine release from astrocytes is a Ca^2+^-dependent mechanism, the source of Ca^2+^ underlying this process remained poorly understood. In agreement with our finding, it was shown few years later that d-serine release was regulated by Ca^2+^ influx in astrocytes through transient receptor potential ankyrin 1 channels (TRPA1; Shigetomi et al. [16].

## d-Serine Release from Astrocytes is Regulated by IP_3_Rs

Another important pathway known to be involved in Ca^2+^ signaling in astrocytes involves inositol 1,4,5-trisphosphate receptors (IP₃Rs) and in particular IP₃Rs subtype 2 (IP₃R2) which is highly expressed in astroglial cells. Experiments carried out in knock-out mice have indicated that hippocampal LTP was unaffected by genetic ablation of IP₃R2 [17]. Although these results prompted the authors to rapidly conclude that astrocytes and gliotransmission were not involved in synaptic plasticity, such findings raised the possibility that another pathway or potentially another subtype of IP₃Rs could be at play in astrocytes. In agreement with this hypothesis, Ca^2+^ imaging experiments performed in IP₃R2 and IP₃R2-3 knock-out mice later revealed that multiple IP_3_Rs subtypes contributed to Ca^2+^ signaling in CA1 hippocampal astrocytes. While global Ca²⁺ signals were abolished in IP₃R2 knock-out mice, localized Ca^2+^ transients in astrocytic processes persisted, emphasizing the spatial complexity of astrocytic Ca²⁺ dynamics [18]. To investigate the potential role of IP₃Rs in LTP induction, the membrane impermeable pan-inhibitor of IP_3_Rs heparin was dialyzed into single astrocytes through the patch pipette. Such an inhibition of all IP_3_Rs subtypes yielded a strong reduction in Ca^2+^ activity within astrocytic processes and significantly impaired LTP. Here again, exogenous d-serine application rescued completely LTP, thereby demonstrating that d-amino acid astrocytic supply to synaptic NMDARs is an IP₃R-dependent mechanism [18]. That synaptic plasticity and memory depends on astrocytic IP3Rs was also established recently [19]. In agreement with our findings, this study showed that chemogenic activation of the Gq pathway specifically expressed in astrocytes was sufficient to induce de novo LTP at CA3-CA1 synapses and to enhance memory acquisition [19]. One very important remaining question regarding synaptic d-serine supply is whether specific receptors on the astrocytic membrane are responsible for regulating the spatial and temporal release of the co-agonist. Among the different putative candidates, we focused on type-1 cannabinoid receptors receptors (CB1Rs).

## Astrocytic CB1 Receptors Control d-Serine Availability, Long-Term Synaptic Plasticity and Recognition Memory

At that time, emerging evidence suggested that endocannabinoids, through the activation of CB1Rs could be involved in this process. Indeed, it has been shown previously that activation of astrocytic CB1Rs is leading to an increase of intracellular Ca^2+^ through G protein–coupled signaling pathways [20]. To assess a possible role for these receptors in d-serine release, we used a mouse model in which CB1Rs were knocked out selectively in astrocytes (GFAP-CB1-KO mice; Robin et al. [21]). In the absence of astrocytic CB1Rs, synaptic availability of d-serine in hippocampal acute brain slices was found to be reduced significantly, resulting in impaired NMDAR activity and LTP at CA3-CA1 synapses [21]. Most importantly, these impairments in NMDAR-dependent activity and plasticity could be rescued with exogenous d-serine. Because novel object recognition (NOR) memory depends on hippocampal activity, we assessed the consequence of knocking out CB1 receptors in astrocytes on NOR task [21]. We observed that not only NOR memory was deficient in these GFAP-CB1-KO mice, but also that exogenous d-serine reverted those memory impairments. Altogether, this study provides compelling evidence that astrocytic CB1Rs control recognition memory by regulating synaptic d-serine availability and, consequently, synaptic NMDARs functions. In agreement with such a role for astrocytic CB1Rs in d-serine release, it has been reported recently that astrocytes could also regulate the threshold and amplitude of dendritic spikes in CA1 pyramidal neurons through the CB1-dependent release of d-serine from astrocytes. Through this process astrocytes contribute to a positive loop between pyramidal cells excitability and dendritic spiking, a process that appears to be important for spatial learning [22]. These results strengthen the idea that cannabinoid signaling is a key factor in the functional architecture of the tripartite synapse and highlight astrocytes as dynamic regulators of cognitive processes. While the activation of membrane receptors like CB1Rs appears to be involved, another key parameter for the supply of d-serine to synaptic NMDARs is the close anatomical proximity between astrocytic processes and synaptic neuronal elements [11]. Notably, signaling crosstalk between astrocytes and neurons can occur through the interaction of cell adhesion molecules (CAMs), which relies on the tight spatial relationship between astrocytes and their synaptic partners. Among these CAMs, the erythropoietin-producing hepatocellular carcinoma (Eph) receptor family [23] and their ligands, ephrins, are recognized for their role in facilitating bidirectional communication between astrocytes and neurons [24, 25].

## Astrocytic EphB3 Receptors Regulate Synaptic d-Serine Availability

Eph receptors and their ephrin ligands are uniquely positioned at the astrocyte-neuron interface and have been implicated in a variety of synaptic processes such as spine morphogenesis, synaptic stabilization, and neurotransmitter receptor trafficking [26]. Interestingly, in astrocytic cultures, the stimulation of EphB3 receptors induced an increase of d-serine in the extracellular medium [27, 28]. In addition, LTP was impaired when either EphB3 receptors or ephrinB3 ligands were knocked out in all cell types [27, 29]. To go further, we investigated the role of astrocytic EphB3 receptors in astrocyte-driven regulation of NMDARs functions from the synapse to NOR memory [30]. To this end we inhibited the endogenous activity of EphB3 receptors, either through a pharmacological approach, or by knocking them down specifically in astrocytes. Both types of manipulations yielded similar results that consisted in a major reduction in d-serine synaptic availability and an impairment of synaptic NMDARs activity including LTP at CA3-CA1 synapses. In addition, we found that NOR memory was impaired in the EphB3R knockdown mice. Here again, all the synaptic and memory deficits were rescued by exogenous d-serine [30]. Taken together, our recent findings indicate that both CB1 and EphB3 receptors regulate NMDARs activity, long-term synaptic plasticity and NOR memory through the control of NMDAR co-agonist-binding site occupancy [21, 30]. Whether there is a direct interaction between these two signaling systems remains an open question that will require additional investigations. Finally, it has also been shown that the amount of d-serine astrocytes provide to the synapse varies as a function of the wakefulness state of the animals [31]. Intriguingly, astrocytes are not only present at the level of synapses, but they are also well known to be at the interface in between blood vessels and synapses. l-serine, the precursor of d-serine, is an astrocytic glycolysis intermediate [32, 33]. Therefore, we then investigated whether metabolism and d-serine gliotransmission were coupled.

## d-Serine, at the Frontier Between Metabolism and Gliotransmission

Interestingly, the early phase of Alzheimer’s disease (AD) is characterized by both metabolic and synaptic impairments. Furthermore, l-serine, the precursor d-serine, is an astrocytic glycolysis intermediate: In the phosphorylated pathway, the glycolytic intermediate 3-phosphoglycerate is first transformed into 3-phosphohydroxypyruvate by the enzyme PHGDH, leading then ultimately to the synthesis of l-serine [33]. To address a possible coupling between metabolism and gliotransmission, we investigated synaptic transmission in the hippocampus of 3xTg-AD mice, a model of AD. We established that the glycolytic flux was impaired in these animals together with the synthesis of both l-serine and d-serine, leading to a decrease supply of synaptic d-serine that resulted in impaired NMDARs activity, LTP, LTD and spatial memory [34]. Importantly while synaptic and memory deficits were rescued by exogenous l-serine or d-serine, these deficits were reproduced when PHGDH was specifically knocked-out in astrocytes, confirming the intimate link between astrocytic metabolism and gliotransmission [34].

Using a different mice model in which CB1Rs are specifically knocked out from astrocytes, we further demonstrated the existence of a coupling between astrocytic glycolysis, gliotransmission, long-term synaptic plasticity and memory [35]. This is based on the discovery that astrocytic CB1 receptor controls the synthesis and release of lactate. Once released, lactate binds to the lactate receptor HCAR1, whose activation promotes l-serine synthesis in the astrocyte, thereby stimulating d-serine production, its release in the synaptic cleft and NMDAR-dependent activity [35]. These findings strengthen the importance of astrocytic metabolic flexibility in maintaining both synaptic functions and cognitive health with lactate and d-serine acting as key players in the intricate relationship prevailing between astrocyte and cognition. While d-serine is the co-agonist of synaptic NMDARs, the identity of the co-agonist of extra-synaptic NMDARs needed to be identified. This, was one of the goals of a study realized in 2012 [13].

## d-Serine and Glycine Gate Synaptic and Extra-synaptic NMDARs Respectively

To tackle the nature of the endogenous co-agonist of synaptic and extra-synaptic NMDARs, we took advantage of the difference in NMDAR subunit composition between synaptic and extra-synaptic location to differentiate these receptors. Whereas synaptic NMDARs at CA3-CA1 synapse are enriched in GluN2A subunit, extra-synaptic receptors do contain GluN2B subunit. Based on this difference in composition and a selective pharmacology, we found that d-serine depletion was strongly impairing synaptic NMDARs activity while glycine depletion only affected the activity of extra-synaptic receptors. Our results indicate that d-serine and glycine are respectively gating synaptic GluN2A-containing NMDARs and extra-synaptic GluN2B-containing NMDARs [13]. Taking advantage of this discovery, we were able to show that d-serine, but not glycine, is required for LTP induction. Homosynaptic LTD on the other hand requires both d-serine and glycine. Taken together these findings suggest that synaptic and extra-synaptic NMDARs are required for LTD induction whereas only synaptic NMDARs are required for LTP. In agreement with our findings, it was reported more recently that d-serine was involved not only in homosynaptic LTD but also in a different form of synaptic plasticity [36]. In this study, the authors showed that d-serine and glutamate, both released by astrocytes through the anion channel BEST1, were playing a key role in æ1adrenergic receptor–dependent heterosynaptic LTD, a process that is also important for metaplasticity and cognitive functions [36]. The subunit composition and synaptic localization of NMDARs are critical determinants of synaptic function and plasticity. In particular, the balance between GluN2A- and GluN2B-containing NMDARs regulates key processes from developmental processes to adulthood. As d-serine and glycine respectively gate GluN2A- and GluN2B-containing NMDARs, we then investigated if they exert distinct effects on NMDARs trafficking and synaptic localization.

## d-Serine Regulates GluN2B-NMDAR Localization and Composition

Using quantum dots tracking, we were able to provide direct evidences indicating that d-serine and glycine had an opposite action on the surface dynamics and synaptic content of GluN2B- and GluN2A-containing NMDARs in hippocampal neurons [37]. While d-serine reduces the presence of GluN2B at the synapse, glycine favors it. The opposite is true for GluN2A with a positive effect of d-serine and an antagonism with glycine. In summary, the presence of d-serine in the cleft would favor GluN2A-containg NMDARs at the synapse while preventing the presence of GluN2B-NMDARs. This, is exactly the situation that is prevailing at CA3-CA1 synapses in adult animals and this, could be due to the segregated location of d-serine and glycine. Finally, the impact of d-serine and glycine on NMDARs subunits composition appears to be developmentally regulated. Indeed, electrophysiological recordings revealed that glycine is the predominant co-agonist of synaptic GluN2B-containing NMDARs from P5 to P10, while d-serine takes over after P10 [37]. This is in complete agreement with our findings on the influence of the two co-agonists on NMDARs composition since at young age NMDARs at CA3-CA1 synapses are enriched in GluN2B before switching to GluN2A-containing receptors later during development. It appears that this switch in subunit that has been well documented in the literature is in fact directly related to the switch from glycine to d-serine as endogenous co-agonist of synaptic NMDARs. These findings revealed an unexpected role for d-serine in the developmental tuning of NMDAR composition at synaptic sites and underscore the broader regulatory influence of astrocyte-derived co-agonists on excitatory synaptic plasticity.

## Conclusion and Perspectives

**Fig. 1:**
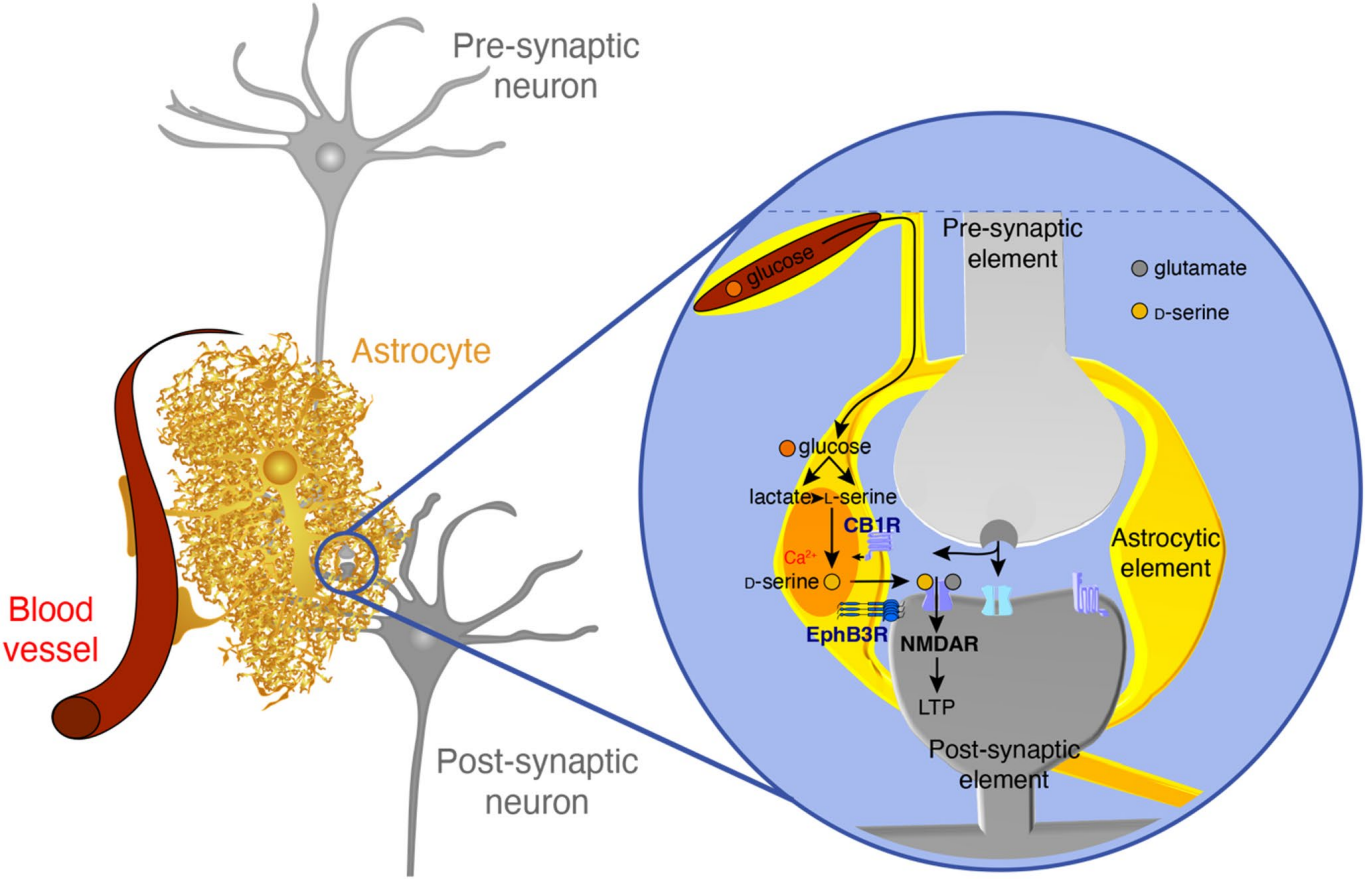
Astrocytic d-serine from its synthesis to LTP induction. Astrocytes synthesise d-serine through a two-step process, beginning with the synthesis of l-serine via glycolysis, that is then converted into d-serine by the serine racemase. Once produced, d-serine is released into the synaptic cleft though specific Ca^2+^-dependent astrocytic mechanisms. The CB1 receptor (CB1R), on astrocytic membranes, plays a key role in controlling both the synthesis and release of lactate, promoting the synthesis of astrocytic l-serine. Importantly, the close apposition of the astrocytic process with its neuronal synaptic partners, allows the interaction of astrocytic EphB3 receptors (EphB3R) with its neuronal ligand ephrinB3, regulating d-serine availability in the synaptic cleft. Once release, d-serine acts as a co-agonist of synaptic NMDARs, whose activation can lead to the induction of long-term synaptic potentiation (LTP), a cellular process involved in memory

Together, these studies present a compelling body of evidence that astrocytes actively regulate synaptic functions and cognitive processes through the synthesis and release of d-serine (Fig. 1). From structural plasticity and transmitter uptake to NMDAR co-agonist availability and intracellular signaling, astrocytes exert multifaceted control over the neuronal environment. In particular, Ca^2+^ signaling in astrocytic processes is now recognized as a crucial trigger for d-serine release involved in NMDAR-dependent synaptic plasticity. These findings not only substantiate the concept of the tripartite synapse but also position astrocytes as dynamic Ca^2+^-sensitive regulators of cognition, and in particular of learning, memory recall and flexibility [19, 22, 36, 38]. By bridging molecular, structural, and functional domains of brain physiology, astrocytic d-serine emerges as a critical mediator of memory, and a promising target for future therapeutic treatment in neurological disorders. It is important to notice that in this review we focused principally on NMDARs functions in the hypothalamus and the hippocampus, but similar data were reported in other brains area including the cortex where LTP and LTD were shown to be dependent upon astrocyte-derived d-serine [37, 38]. This includes LTP induced by high-frequency stimulation as well as low frequency stimulation-induced LTD and spike timing-dependent LTD.

Although this review primarily focuses on astrocytic d-serine as a crucial co-agonist for NMDARs, we acknowledge the ongoing debate regarding the relative contributions of astrocytes vs. neurons in its production and release. While a substantial body of evidence supports astrocytes as the principal source of d-serine in several brain regions, reports of neuronal synthesis and release highlight the complexity of this issue. The presence of serine racemase and d-serine in astrocytes [9, 39] and neurons [40–42] has opened a debate on the respective role of d-serine from astrocytes and neurons in NMDARs functions. Indeed, as astrocytes have the capacity to release both l-serine and d-serine, it has been proposed that l-serine from astrocytes could be uptaken by neurons to be converted into d-serine. Such d-serine could be in turn released to regulate the activity of synaptic NMDARs [43]. Interestingly, while these authors considered that d-serine from neurons could be transported back to astrocytes to be then released, they showed that when SNARE-dependent exocytosis was inhibited specifically in astrocytes, LTP induction was impaired. While exogenous d-serine rescued LTP, l-serine had no impact [44]. This confirms that d-serine released from astrocytes is necessary for LTP induction [44]. Interestingly, while chronic depletion of serine racemase in neurons did not change the synaptic level of d-serine, the subunit composition of synaptic NMDARs was altered [45]. Therefore, it is intriguing to consider that d-serine released from astrocytes and neurons could have distinct roles [18, 43]. Future experiments would be essential to elucidate the distinct roles and mechanisms by which both astrocytic and neuronal d-serine contribute to synaptic functions and cognitive processes. A deeper understanding of this interplay is critical for a comprehensive view of d-serine’s impact on memory. One promising direction involves the development of genetically encoded d-serine sensors with high sensitivity and temporal resolution, enabling real-time tracking of d-serine, at tripartite synapses during memory tasks [46]. In addition, coupling these sensors with optogenetic silencing of specific astrocyte populations could clarify how astrocytic d-serine regulates NMDAR-dependent long-term synaptic plasticity in vivo. Finally, astrocyte-specific manipulations of serine racemase in defined brain regions, such as the hippocampus or prefrontal cortex, combined with behavioral assays assessing memory, would help bridge molecular mechanisms with cognitive outcomes.

## Data Availability

No datasets were generated or analysed during the current study.
